# Evaluation of Common Prosperity Level and Regional Difference Analysis along the Yangtze River Economic Belt

**DOI:** 10.3390/ijerph191911851

**Published:** 2022-09-20

**Authors:** Yuhan Wang, Zenghui Huo, Dongpo Li, Mei Zhang

**Affiliations:** 1 College of Economics & Management, China Jiliang University, Hangzhou 310018, China; 2 College of Economics and Trade, Hunan University of Technology and Business, Changsha 410205, China; 3 College of Economics & Management, Zhejiang University of Water Resources and Electric Power, Hangzhou 310018, China

**Keywords:** Yangtze River Economic Belt, common prosperity, TOPSIS, regional differences

## Abstract

Common prosperity is the essential requirement of socialism as well as the common aspiration of social people. This article constructed an evaluation index system of 25 indicators for common prosperity, covering four dimensions of material wealth, harmonious social life, rich spiritual life, and livable ecological environment. The TOPSIS method was used to comprehensively rank nine provinces and two municipalities in the Yangtze River Economic Belt. The results show that the level of common prosperity along the Yangtze River Economic Belt increased significantly from 2010 to 2019, and the level of common prosperity in the lower reaches of the Yangtze River Economic Belt is much higher than that in the middle and upper reaches. According to the differences in common prosperity levels among regions, provinces and cities are divided into three categories: high, unbalanced, and low. Combined with the characteristics of each type of region, policy suggestions were put forward from the perspectives of strengthening the regional industrial cooperation mechanism, deepening the construction of regional livelihood infrastructure and basic public services, and improving the ability of regional environmental coordination.

## 1. Instruction

On the occasion of the centenary of the founding of the Communist Party of China (CPC), China has won an overall victory in the battle against poverty. All the 98.99 million rural residents living below the current poverty line have been lifted out of poverty. A total of 832 impoverished counties and 128,000 impoverished villages have been lifted out of poverty and this marks the completion of the arduous task of eradicating absolute poverty. Since 2012, more than 10 million people have been lifted out of poverty every year, and the income level of the impoverished population has risen significantly. All of them have achieved the goal of poverty reduction set in the UN 2030 Agenda for Sustainable Development, 10 years ahead of schedule [1]. With the rapid development of China’s economy, the gap between urban and rural areas and regions is becoming increasingly obvious, which affects China’s economic development and social harmony and militates against the goal of common prosperity. Improving the income level of poor areas and addressing the gap between the rich and the poor is an inevitable choice to achieve common prosperity [2].

The Yangtze River Economic Belt covers the provinces of Shanghai, Jiangsu, Zhejiang, Anhui, Jiangxi, Hubei, Hunan, Chongqing, Sichuan, Yunnan, and Guizhou. In 2020, the total population of this region was about 610 million, and its GDP was about 47 trillion yuan, accounting for more than 40% of the total in China. As one of the country’s three major development strategies, the CPC’s Central Committee, with President Xi Jinping at its core, has made major plans for the development of the Yangtze Economic Belt on many occasions. On 15 November 2020, President Xi Jinping presided over the Symposium on the Development of the Yangtze River Economic Belt, stressing that the Yangtze River Economic Belt will become the main battlefield of China’s ecological priority and green development, will smoothen the arteries of the domestic and international double circulation, and lead high-quality economic development [3]. Compared with the other regions, the Yangtze River Economic Belt has prominent advantages in economic development, reform and innovation, and ecological improvement. However, economic development is still a big difference [4]. The allocation of educational resources is unbalanced [5] and scientific competitiveness is developing disproportionately [6]. Contradictions between the economy, resources, and the environment [7] and unbalanced and inadequate development are prominent problems. Arguably, narrowing the regional differences is the only way to promote the high-quality development of the region and the basic premise of achieving common prosperity. Therefore, the measurement and characterization of the degree and characteristics of common prosperity along the Yangtze River Economic Belt will provide theoretical guidance and policy suggestions for promoting high-quality regional development and achieving common prosperity in an all-round approach.

As China enters the new era, the principal social contradiction and actual development situation are also changing. From a macro perspective, the unbalanced development between regions and urban and rural areas is increasingly hindering economic growth. Microscopically, the generation of the income gap between regional residents is closely related to the primary distribution and redistribution, and common prosperity has Chinese characteristics to solve the current income inequality and regional disparities in China’s road and the fundamental policy, then how to define regional common prosperity? How to measure the development level of common prosperity in a certain region? This is the problem to be solved in this paper. Therefore, the purpose of this study includes two aspects. The first is to measure and characterize the degree of common prosperity and its regular characteristics along the Yangtze River Economic Belt and quantify common prosperity to provide new research directions. The second is to provide theoretical guidance and policy recommendations for promoting high-quality development in the region and moving towards common prosperity in an all-round way.

The rest of the study is arranged as follows: Section 2 is a review of the relevant literature at home and abroad, including the evolution and development of common prosperity and its practical implications; Section 3 introduces the research area and research methods; Section 4 analyzes the empirical results; Section 5 discusses and summarizes the study.

## 2. Literature Review

### 2.1. Policy Evolution

The concept of common prosperity first appeared in the theory of scientific socialism. Marx believed that the development of production in the future socialist society would be “aimed at the prosperity of all people”, and that common prosperity contains the qualities of both productive forces and relations of production [8]. Liu Tongfang pointed out that common prosperity requires the liberation and development of the productive forces so as to create a material foundation for the highly developed productive forces and the full flow of material wealth. Common prosperity is a state of equalization and equality in the form of social members’ possession of social wealth such as means of production and living and is a historical product of the development of productive forces and social progress [9]. From the perspective of historical development, the gap between the rich and the poor is not a common phenomenon existing in all social and historical stages but is closely related to the development of productive forces and the changes of ownership forms brought about by it. Marx thought that new productive forces and production relations did not appear out of thin air or subjective imagination to construct the result. However, with the development of productivity, as well as with the traditional system of ownership relations within their opposite generated, the performance of common prosperity is fundamentally productivity development and the change of ownership relationship created by the result of [10].

As the principle and goal of correcting the social gap between the rich and the poor, the actual appearance of common prosperity has social and historical roots and historical inevitability [11].

The concept and connotations of common prosperity are constantly evolving. After the founding of New China, Mao Zedong was the first to clearly put forward the concept of common prosperity, “so that the peasants can gradually and completely get rid of the condition of poverty and achieve a life of common prosperity with the view to ensure and general prosperity” [12]. In the practice of reform and opening up, Deng Xiaoping realized that the average sharing of social wealth could not stimulate social vitality, and that it was necessary to allow some people and some regions to get rich first so as to drive the latter to get rich first, and on this basis, he clarified the essence of socialism [13]. With the establishment of the socialist market economy system, Jiang Zemin emphasized the need to achieve common prosperity under the premise of “giving priority to efficiency and taking into account equity” [14]. With the problem of unbalanced development becoming increasingly prominent, Hu Jintao proposed balancing equity and efficiency, and put equity in a more prominent position so that all people can share the fruits of development [15].

Entering the new era, Xi Jinping’s understanding of common prosperity reflects the new characteristics of the times. Xi has repeatedly stressed that “taking poverty alleviation as the top priority and lifting all the rural poor out of poverty under the current standards is a major measure to promote the common prosperity of all the people” [1]. He embraced the “two-step” strategy as the path to achieve common prosperity. In the following, he put forward the concept of shared development, and inherited and developed the concept of common prosperity. The essence of the shared concept is to adhere to the concept of people-centered development, which reflects the requirement of gradually realizing common prosperity.

### 2.2. The Meaning of Common Prosperity

At the tenth meeting of the Central Finance and Economics Commission in 2020, Xi Jinping stressed that common prosperity is the common prosperity for all people, the prosperity of both the material and spiritual life of the people, not the prosperity of a few people, nor the neat and tidy egalitarianism. He emphasized that common prosperity should be promoted in stages. The “Opinions of the CPC Central Committee and the State Council on Supporting Zhejiang’s High-Quality Development and Building a Common Wealth Demonstration Zone” further pointed out the connotation of common wealth. They stated that “Common wealth has distinctive features of the times and Chinese characteristics, and is the common wealth of all people who, through hard work and mutual help, generally achieve a rich and affluent life, self-confidence and self-improvement in spirit, a livable and pleasant environment, a harmonious society, and universal public services. Through hard work and mutual help, all people can generally achieve a rich and prosperous life, self-confidence and self-improvement, a livable and pleasant environment, social harmony, and universal public services, achieve comprehensive human development and social progress, and share the fruits of reform and development and a happy and beautiful life” [16].

Researchers have defined the notion of common wealth from a multidimensional perspective: common wealth is a measure of the way and level of social wealth possession by a country or society and its members [17]. Liu Peilin believes that common prosperity means a multidimensional and integrated happy life and comprehensive human development [18]. In the same vein, Yu suggests that common prosperity is the opportunity and ability for all people to participate equally in high-quality economic and social development and share the fruits of economic and social development by compensating and correcting inequalities caused by systemic factors [19]. Zhang Laiming pointed out that common prosperity does not only seek to promote fair income distribution, but also to focus on promoting the equalization of basic public services, equal opportunities, health equity, spiritual civilization, and cultural resources for all, and the overall development of people and social progress [20]. Shi Jinchuan, on the other hand, analyzes common prosperity from the theoretical perspective of modern economics, involving the Pareto improvement and the Kaldor–Hicks improvement in economics. Although the Pareto improvement itself does not involve the distribution of income and wealth, it is closely related to the concept of affluence in common prosperity from the perspective of the efficiency of resource allocation and the increase of income and wealth in society. From the perspective of economic theory, the Kaldor–Hicks improvement is the problem of income redistribution. The compensation mechanism involved in the Kaldor–Hicks improvement mainly aims at the income increment in the social and economic development process, that is, the redistribution problem of the incremental piece after the cake becomes larger [9].

The reality of income inequality among the population has likewise emerged or is being experienced in developed countries and regions in the course of development. Fabrizio Germano takes a new entropy-based macro evolutionary approach to address the study of why societies vary so much in terms of inequality and why different levels of inequality persist so long in societies, and suggests that societies with more balanced and reciprocal interactions lead to more income equality and a non-stratified society. In contrast, societies with unbalanced and non-reciprocal interactions lead to more inequality and a potentially stratified society [21]. Ilona Reindl and Jean-Robert Tyran experimentally studied popular support for economic inclusion, that is, policies of equal income-generating opportunities, and discuss how this support is influenced by the future income redistribution [22]. In addition, foreign scholars often use the concept of happiness to measure whether the development of a certain region or country is balanced, and the income gap of residents. Heinz Welsch and Udo Bonn used the European Union in the 1990s as a research context to study the convergence of quality of life among EU member states using a measure of subjective well-being. They use self-rated life satisfaction elicited from large-scale surveys as an empirical approximation of quality of life. Conducted regression analysis to investigate the relationship between life satisfaction and macroeconomic indicators, namely per capita income, unemployment, and inflation, and found that the convergence of life satisfaction across EU member states can be attributed, to a large extent, to the convergence of macroeconomic conditions. In contrast, among various macroeconomic indicators, the convergence of inflation plays an important role in the convergence of life satisfaction [23].

### 2.3. Measure of Common Prosperity

Although there are differences in the selection of indicators for measuring common prosperity, the existing studies have basically the same construction concept. Some studies measure common prosperity based on two dimensions: the overall affluence and the degree of sharing development achievements. Liu Peilin constructed an indicator system framework containing two dimensions: the overall affluence and the degree of sharing development achievements [18]. Wan Haiyuan, among others, used the national income per capital to measure the level of development, the Gini coefficient of disposable income per capital to reflect the degree of social sharing, and constructed an equal-weighted common wealth function relationship equation to quantify the degree of common wealth [24]. Some studies have further expanded the indicators of common prosperity measurement: for instance, Jiang Yongmu, among others. Theoretically, a common prosperity indicator system was constructed with four dimensions: peoplehood, sharing, development, and security [25]. Yang Yiyong, among others, considered common wealth as the organic unity of common and affluent, and designed an indicator system including economic, cultural, ecological, rule of law, public service, and other factors [26].

According to the meaning of “common prosperity” summarized in the previous chapter, “prosperity” is not only the growth and satisfaction of material wealth, but also the concept of sustainable development and the “community of human destiny” proposed by China. According to the concept of sustainable development and the concept of “community of human destiny” proposed by our country, taking into account the current global warming and environmental deterioration, the level of common prosperity should also include indicators related to the rational use of renewable resources and the development of green industries in the study area. Based on the awareness of climate change and its consequences, many international institutions and countries have made sustainable development a central goal to facilitate the process of transition from an energy economy to a low-carbon economy, a process that requires increasing the share of renewable energy in the energy mix [27]. In this regard, China has also proposed a goal for China to achieve peak carbon by 2030 and carbon neutrality by 2060 [28]. In their study, Rosa Duart et al. suggested that the infrastructure required for the use of renewable energy sources is usually set up in rural areas, and they retrospectively analyzed the compatibility of rural development and environmental objectives using a synthetic control method, using wind energy as an example, showing that the compatibility of socioeconomic, demographic, and environmental objectives may be difficult to achieve in rural areas, with negative effects in terms of rural population, and can only create temporary employment opportunities. Therefore, wanting to reduce the income gap and alleviate the development imbalance, it is necessary to link to the territory and its population in this aspect of renewable energy use in order to achieve a just energy transition [27]; Yan Haijuan and Hu Xiaofei et al. believed the Yangtze River Economic Belt is an important ecological security barrier and energy base in China, and has become part of the national ecological protection and high-quality development strategy [29,30], so the ability to rationally develop and utilize renewable resources and the level of low-carbon economic development are important dimensions for examining the level of common wealth development in the Yangtze River Economic Belt region.

In terms of measurement methods, based on relevant research, Chen Lijun et al. used hierarchical analysis to test the weights of the constructed common wealth index model, but there is a certain subjectivity in their judgment process [31]. Shen Yun et al. used the entropy TOPSIS method to judge the common prosperity of rural residents in China [32]. Some other studies have used the entropy-weighted TOPSIS method to analyze regional differences in different dimensions [33,34]. This article believes that the entropy TOPSIS method can make up for the shortcomings of the hierarchical analysis method in the research process and is more objective and accurate in the measurement of indicators.

The existing studies have produced rich results in terms of index construction and theoretical analysis, providing a good theoretical basis for future in-depth research on common prosperity. However, there are still some weaknesses. Firstly, the research focuses on economic development and lacks careful consideration of the political, ecological, cultural and health factors. Secondly, although regional development in China presents large differences, and the realization of common prosperity should be achieved by point to point, there is still a lack of examination of the regional common prosperity. Based on this, this article constructs regional common prosperity indicators from four dimensions: material life, social culture, spiritual life, and ecological environment. In addition, it measures the level of regional common prosperity along the Yangtze River Economic Belt using the entropy-weighted TOPSIS method, and explores the different characteristics, sources, and common prosperity patterns of regional common prosperity.

## 3. Regional Common Wealth Evaluation Index System

### 3.1. Survey Region

**Figure 1:**
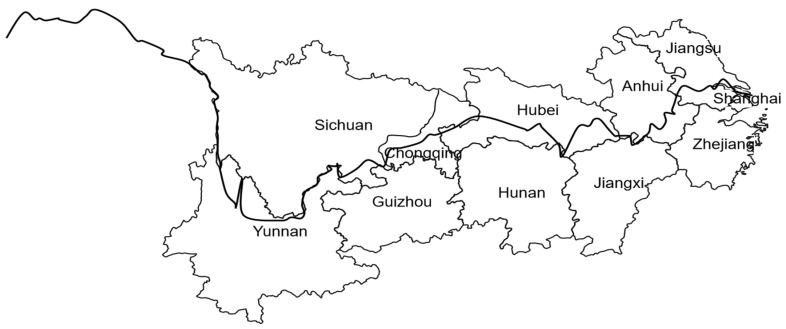
Location map of Yangtze River Economic Belt.

As an important economic belt of China’s “Belt and Road” strategy, the Yangtze River Economic Belt is one of the regions with the strongest comprehensive strength and the most important strategic support role [3]. Figure 1 shows it spans three major regions in the east, middle, and western parts of China, covering 11 provinces and cities in Shanghai, Jiangsu, Zhejiang, Anhui, Jiangxi, Hunan, Hubei, Chongqing, Sichuan, Guizhou, and Yunnan. This is shown in Figure 1. Due to its unique geographical location, important ecological environment, and huge development potential, the Yangtze River Economic Belt occupies a pivotal position in China’s economic landscape. Promoting the coordinated development of the Yangtze River Economic Belt region is of great significance in solving the problem of unbalanced and inadequate regional development with the view to achieve common prosperity in the region.

### 3.2. Measurement Index

Common prosperity is the essential requirement of socialism and the greatest superiority of socialism. Common wealth is shared wealth among all people; comprehensive wealth and gradual wealth. The former reflects the level of economic and social development, while the latter reflects the process of fair sharing of development opportunities and fruits by all people. In terms of content, the essence of common prosperity is to meet the growing needs of the people for a better life in all aspects. Common prosperity should meet the people’s needs for a good material and spiritual life. This is consistent with General Secretary Jinping Xi when he said that common prosperity is the affluence of both the material and spiritual life of the people [35]. Common prosperity should also meet people’s needs for good basic public services and a good livable living environment. Based on the existing research, this article proposes four dimensional indicators for evaluating regional common prosperity: material life affluence, social life affluence, spiritual life affluence, and livable ecological environment.

Affluent material life reflects the regional economic development level, residents’ income and consumption gap, and the degree to which the regional economic development level satisfies the people’s good material needs. Among them, the ratio of per capital GDP and the added value of the tertiary industry to GDP represent the level of regional economic development. The ratio of research and experimental development (R&D) expenditure to GDP and the urbanization rate represent the regional scientific and technological strength and urbanization level. On the other hand, the consumer price index, Engel ss coefficient, and urban and rural residents’ disposable income difference indicate the regional residents’ consumption quality and income gap.

Harmonious social life reflects the stable and harmonious situation of social life in the region, such as inclusive and equal social security, sufficient employment, adequate housing, and a convenient transport system. It reflects the degree to which regional social development meets the needs of the people for good basic public services. Among them are the basic pension insurance participation rate, the number of beds in medical institutions per 1000 permanent residents, the basic medical insurance coverage rate, and the number of health technicians per 1000 permanent residents. These measures indicate that regional social security benefits are inclusive. Level of equality, urban registered unemployment rate, per capital housing area of urban and rural residents, the number of family cars per 100 households in urban areas, and the number of family cars per 100 households in rural areas represent indicators such as regional employment status, living conditions, and traffic convenience.

Prosperity in spiritual life reflects the level of people’s shared public cultural services and spiritual achievement. This reflects the contribution level of regional cultural resources to meeting people’s spiritual needs and enriching people’s spiritual life. Among them are the proportion of education expenditure in general public budget expenditure and the proportion of urban and rural residents’ education. Culture and entertainment expenditure represent regional education investment, residents’ education, culture and entertainment consumption level, the number of teachers per 1000 resident population, and the number of college students per 1000 resident population.

The livable ecological environment reflects the level of human settlements and ecological livability such as regional ecological protection and environmental governance. It also reflects the satisfaction of regional environmental governance and protection for the people’s needs for a beautiful ecological environment. Among them are the green coverage rate of built-up areas, per capital park green space, and forest coverage rate. All the indicators show the degree of regional ecological protection and ecological balance. The sewage treatment rate and the harmless treatment rate of domestic waste indicate the level of regional sewage and domestic waste treatment. The specific index content and index nature are shown in Table 1.

### 3.3. The Data Source

The data on the indicators of economic prosperity and social harmony involved in this article are mainly from the provincial statistical yearbooks of 11 provinces and cities from 2010 to 2019. The number of pension insurance participants and the number of medical insurance participants are from the 2010–2019 China Labor Statistics Yearbook. The index data of education, science, culture, health, and ecological civilization mainly come from the 2010–2019 China Statistical Yearbook, and the sewage treatment rate index data come from the 2010–2019 China Urban Construction Statistical Yearbook and the provincial statistical yearbooks from 11 provinces and cities. At the same time, the author calculated the specific indicators involved in the calculation of common prosperity from the original data.

### 3.4. Research Methods

The TOPSIS method is a sorting technique that approximates the ideal solution, and sorts by detecting the closeness of the evaluation object to the optimal solution and the worst solution [32]. It can effectively solve the multi-objective decision-making problem and facilitate each evaluation objects’ horizontal and vertical comparative analysis [36]. When determining the index weight, the entropy weight method is usually used to calculate weight. Dariusz Kacprzak, specializing in multicriteria decision-making methods for ordered fuzzy data, mentioned that the TOPSIS method allows better differentiation of alternatives than the classical simple summation weighting (SAW) method, and that in practical decision problems, most multi-objective decision models use only subjective weights defined by the decision maker. When reliable subjective weights are not available, objective weights become useful when reliable subjective weights are not available, and one of the ways to obtain objective weights is to apply the concept of entropy [37]. Dariusz Kacprzak also extended the TOPSIS method to group decision-making, where the algorithm first determines the weights of the decision makers and then calculates the aggregated decision matrix of all group decision matrices provided by the decision-makers [38].

#### 3.4.1. Build a Standardized Decision Matrix

Assuming that for *m* evaluation areas and *n* evaluation indicators, a data matrix of order *m* × *n* is formed $X=(x_{ij})(i=1,2,\ldots ,m;j=1,2,\ldots ,n)$. For the 2010 data, the initial matrix is an evaluation matrix of order 11 × 25. The evaluation index includes cost type and benefit type. The linear dimensionless method is used to obtain the dimensionless matrix.

(1) $$\mathrm{Cos}t\;\mathrm{index}:\;T_{ij}=\;{\left[t_{ij}\right]}_{m\times n,}t_{ij}=\frac{\max(x_{ij})-x_{ij}}{\max(x_{ij})-\min(x_{ij})}$$

(2) $$\mathrm{Benefit}\;\mathrm{indicators}:\;T_{ij}={\left[t_{ij}\right]}_{m\times n,}t_{ij}=\frac{\max(x_{ij})-x_{ij}}{\max(x_{ij})-\min(x_{ij})}$$

#### 3.4.2. Calculate the Weights of Indicators for Each Year

When the degree of variation of the JTH index is greater, its entropy value $e_{j}$ is smaller, the greater the role of the index in the evaluation of the common wealth index, the greater the corresponding weight. Taking 2019 as an example, the weights of each indicator of the common wealth index of the regions along the Yangtze River Economic Belt are calculated, as shown in Table 2.

The entropy value of each indicator:

(3) $$e_{j}=\frac{-1}{\ln m}\sum_{j=1}^{m}f_{ij}\ln f_{ij}$$

In this

(4) $$f_{ij}=\frac{t_{ij}}{\sum_{j=1}^{m}t_{ij}}$$

Redundancy of each indicator:

(5) $$d_{j}=1-e_{j}$$

Weight of each indicator:

(6) $$w_{j}=\frac{d_{j}}{n-\sum_{j=1}^{m}d_{j}}$$

#### 3.4.3. Determine the Optimal Solution and the Worst Solution

Find the ideal solution:

(7) $$S^{+}=(S_{1}^{+},\ldots ,S_{j}^{+}),j=1.2....,n\;S_{j}^{+}=\min\{t_{ij}\},i=1,.\ldots ,m$$

Find the negative solution:

(8) $$S^{-}=(S_{1}^{-},\ldots ,S_{j}^{-}),j=1,2,\ldots ,n\;S_{j}^{-}=\max\{t_{ij}\},i=1,\ldots ,m$$

#### 3.4.4. Calculate the Relative Closeness of Each Region to the Positive and Negative Ideal Solutions

The distance between the evaluated area and the positive ideal solution:

(9) $$d_{i}^{+}=\sqrt{\sum_{j=1}^{n}{\left(t_{ij}-t_{j}^{+}\right)}^{2}}$$

The distance between the evaluated area and the negative ideal solution:

(10) $$d_{i}^{-}=\sqrt{\sum_{j=1}^{n}{\left(t_{ij}-t_{j}^{-}\right)}^{2}}$$

The relative closeness of the evaluated area to the ideal solution:

(11) $$D_{i}=\frac{d_{i}^{-}}{\left(d_{i}^{+}+d_{i}^{-}\right)},i=1,2,\ldots ,m$$

The value of $D_{i}$ is between 0 and 1. The larger $D_{i}$ is, the higher the common wealth level of the evaluated area. Each evaluated area is statically sorted according to the value of $D_{i}$, and the area with a larger value of $D_{i}$ is ranked higher. According to the above steps, the common wealth index and ranking results of each province and city are obtained after calculation by software R, as shown in Table 3.

## 4. Comprehensive Diagnosis of Differences in Common Wealth Levels along the Yangtze River Economic Belt

### 4.1. Overall Level Trend Analysis

Table 3 shows that the level of common prosperity in the regions along the Yangtze River Economic Belt had an upward trend during 2010–2019, but there is still a large gap in the level of common prosperity among provinces and cities. Zhejiang, Jiangsu, and Shanghai have higher levels of common prosperity, while Jiangxi, Yunnan, and Guizhou have lower levels, and other provinces have intermediate levels of common prosperity. In particular, the common prosperity levels of Zhejiang, Jiangsu, and Shanghai are relatively stable, always ranking among the top three, and the common prosperity index is higher than the average level of the regions along the Yangtze River Economic Belt. It can be seen that they are the leading examples of common prosperity in the Yangtze River Economic Belt, and the promoters of “the first rich, then rich”. Jiangxi, Yunnan, and Guizhou, who were ranked the last three, have a common prosperity index lower than the regional average. Despite their rapid economic, social, cultural, and ecological development in the past 10 years, their common prosperity level has been limited, and their regional ranking remains unchanged. The difference in the common prosperity evaluation index among Chongqing, Sichuan, Hunan, Anhui, and Hubei is small, but there is still a certain gap with the three provinces and cities in the lower reaches of the Yangtze River.

### 4.2. Analysis of Common Wealth Difference Characteristics

Geographically, the Yangtze River Economic Belt can be divided into three major regions, including the upstream region (Sichuan, Chongqing, Guizhou, and Yunnan provinces), the midstream region (Hubei, Hunan, Jiangxi, and Anhui), and the downstream region (Shanghai, Jiangsu, and Zhejiang). The results show that the common prosperity level of the regions along the Yangtze River Economic Belt is characterized by a high level in the east and a low level in the west. The common prosperity level in the downstream region is much higher than the level in the middle and upstream regions, and the common prosperity level in the upstream region is close to that in the middle region. Table 4 shows that the average value of the common prosperity index in the downstream region ranged from 0.542–0.631, the common prosperity index in the upstream region ranged from 0.244–0.311, and the common prosperity index in the midstream region ranged from 0.264–0.352 during 2010–2019. However, the common prosperity index in the midstream region exceeded that in the upstream region between 2017–2019. The three provinces and cities in the downstream region have the top three common prosperity levels in the entire Yangtze River Economic Belt, far exceeding the regional average. In contrast, among the middle and upstream regions, only Sichuan and Chongqing have above-average common prosperity levels, while all other provinces and regions have common prosperity levels below the regional average.

### 4.3. Analysis of Factors Influencing the Variability of Regional Common Wealth

The regions are ranked in four dimensions, including material affluence, social life harmony, spiritual affluence, and ecological environment livability (see Table 5, Table 6, Table 7 and Table 8), which helps to reveal the sources of the formation of the variability of common prosperity in different regions. From the indicators of the four dimensions, there are structural differences in the levels of common prosperity among regions.

#### 4.3.1. Analysis of Material Life Affluence Factors

The results in Table 5 show that Shanghai, Jiangsu, and Zhejiang rank among the top three in the region in terms of material affluence. As an international metropolis and the most economically developed city in the Yangtze River Delta region, Shanghai, with a Ci value of 0.985, dominates with an absolute advantage. The reason for this is that in 2019, Shanghai’s GDP per capital (157,279 yuan), R&D expenditure ratio (4%), tertiary industry value added ratio (72.56%), and urbanization rate (88.3%) were all leading, 1.46 times, 1.49 times, 1.33 times, and 1.26 times higher than Zhejiang’s, respectively. Zhejiang’s material affluence advantage is that the income gap between urban and rural residents is smaller than in Jiangsu. The proportion of tertiary industry is higher, with the difference between urban and rural residents’ disposable income times in 2019 about 11% lower than that of Jiangsu. The proportion of added value of tertiary industry is higher than that of Jiangsu by 7%, the disadvantage is that the per capital GDP is about 13% lower than that of Jiangsu. In addition, the material affluence levels of Hubei and Chongqing are close to the average (0.326). However, there are also structural differences between them: Hubei’s R&D expenditure ratio and resident income multiplier indicators are better than the regional average, but the tertiary industry share and GDP per capital are lower than the regional average. On the other hand, Chongqing only has an urbanization rate higher than the regional average. Finally, Guizhou and Yunnan have the lowest level of material affluence, with Ci values of 0.060 and 0.050, respectively, which are much lower than the regional average. This is because Yunnan’s GDP per capital, R&D expenditure ratio, and urbanization rate lagged behind across the board in 2019, with only 61%, 46%, and 79% of the regional average.

#### 4.3.2. Harmony Factor Analysis of Social Life

The results in Table 6 show that Jiangsu and Zhejiang have the top two social life harmony factors, with Ci values of 0.839 and 0.668, respectively. This is because Jiangsu leads in resources such as health technicians and beds in nursing institutions, and is more balanced in other social resources. In contrast, Zhejiang leads in the overall rate of pension participation, unemployment rate, and housing area for urban and rural residents, and is slightly under-resourced in terms of beds in medical institutions. Sichuan, Hunan, and Hubei have above-average social life harmony factors (0.423), with Ci values distributed between 0.477 and 0.631. The high registered unemployment rate, low per capital health care resources, and elderly institutions are the main reasons for the low ranking of Shanghai’s social life harmony factor, with their health infrastructure resources, elderly institution resources, rural residents’ car ownership, and residential housing areas significantly behind other regions, resulting in the last two places of Guizhou and Chongqing’s social life harmony factor.

#### 4.3.3. Factor Analysis of Spiritual Life Abundance

Table 7 shows that Jiangsu and Sichuan rank in the top two regarding spiritual life affluence factor, with Ci values of 0.872 and 0.678, respectively. Jiangsu had higher educational resources, such as the number of teachers and college students than other provinces and cities in 2019, reflecting its leading advantage in human educational resources. Sichuan has apparent advantages in terms of the share of education expenditures and college students. Hunan, Hubei, and Anhui have an above-average spiritual life affluence factor (0.471), with Ci values distributed between 0.527 and 0.665. Hunan residents have a higher share of education, culture, and entertainment expenditures, and the ratio of education expenditures to public budget and college students are above average, which leads to Hunan’s leading spiritual life affluence. Hubei has the advantage of more colleges and universities and more college students. Anhui province is above average in terms of education expenditure investment, the number of teachers and college students, among others. Chongqing and Shanghai are in the bottom two positions in terms of spiritual life affluence factor, with Ci values of 0.204 and 0.081, respectively. Shanghai’s low percentage of public budget expenditure on education and the least resources, such as the number of teachers and college students, are the main reasons for this position.

#### 4.3.4. Analysis of Livable Factors of Ecological Environment

Table 8 shows that Chongqing has the highest ecological environment livable factor with a Ci value of 0.831, thanks to the advantages of its green indicators such as green coverage of built-up areas, park green areas, and forest coverage. Zhejiang and Shanghai have a higher ecological environment livable factor than the regional average (0.482), while Jiangxi, Jiangsu, and Guizhou have a slightly lower ecological environment livable factor than the regional average, with Ci values distributed between 0.441 and 0.473. The low forest greening rate is the main reason why Sichuan and Anhui ranked as the last two in terms of ecological environment livable factor.

#### 4.3.5. Types of Regional Common Prosperity

Based on the analysis of the four major factors and on the factor ranking, the 11 provinces and cities are divided into: high-level common wealth regions, uneven common wealth regions, and low-level common wealth regions (see Table 9). (1) Zhejiang, Jiangsu, and Hubei belong to the high level of common wealth. Zhejiang and Jiangsu have a balanced and high level of development in four dimensions, including material affluence, social harmony, spiritual affluence, and ecological livability, and play a benchmark role in the development of common prosperity along the Yangtze River Economic Belt. Meanwhile, Hubei, located in the middle reaches of Yangtze River, is the strategic pivot point of the rise of central China—its common prosperity level ranks first in the middle reaches of the region, and its four factors are in a balanced state. (2) Yunnan, Jiangxi, and Guizhou belong to the low level of common prosperity region. Jiangxi and Yunnan rank in the bottom two for common prosperity due to their lagging in material affluence, social harmony, and spiritual affluence. At the same time, Yunnan has apparent deficiencies in material affluence and ecological livability, and lags behind in social harmony and spiritual affluence. (3) Other provinces and cities belong to the uneven common wealth region. Thanks to its leadership in material affluence and ecological livability, Shanghai ranks third in the region, but its shortcomings in social harmony and spiritual affluence are prominent. In contrast, Sichuan and Hunan are relatively ahead in terms of social harmony and spiritual affluence, but lag behind in terms of material affluence and ecological livability. Meanwhile, Anhui ranks in the middle rank in material affluence, social harmony, and spiritual affluence. However, it has shortcomings in ecological livability. Chongqing has a high level of ecological livability but lags behind in social harmony and cultural affluence. In a nutshell, these provinces and cities still have more obvious shortcomings in the level of common prosperity and belong to the uneven common prosperity.

## 5. Conclusions

Focusing on regional common prosperity measurement, this article constructs a regional common prosperity evaluation index system along the Yangtze River Economic Belt in terms of the dimensions of material affluence, social life harmony, spiritual affluence, and ecological environment livability. The entropy weighting method was applied to determine the index weights, and the TOPSIS method was used for comprehensive ranking. Based on the research of domestic and foreign scholars and the experimental results of this paper, the evaluation system constructed in this study is reasonable and meaningful.

The results show that the level of common prosperity in the regions along the Yangtze River Economic Belt had an upward trend during 2010–2019 and showed prominent regional characteristics of high in the east and low in the west, with the level of common prosperity in the downstream region much higher than the level in the middle and upper reaches. That is to say, the downstream regions of the Long Economic Belt, which have innate advantages such as talents and well-developed coastal transportation and industrial basis, lead the development in all aspects and enjoy a relatively higher degree of common prosperity. On the other hand, the results of this study further prove that the “prosperity” in “common prosperity” involves all aspects of social development and people’s life, not only material income, but also the improvement of population quality, the enrichment of cultural life, the harmonious development of human and natural environment, and the coordinated development of renewable resources’ utilization and industrial upgrading.

From the composition dimension, each region has obvious structural differences in the level of common prosperity. Based on the evaluation results, the regions are classified into high-level common prosperity regions, uneven common prosperity regions, and low-level common prosperity regions. Accordingly, this article proposes the following recommendations.

Improve the income distribution system. First of all, always adhere to the basic distribution system of multiple distribution methods mainly based on the distribution of labor; secondly, the initial distribution and redistribution systems should be complementary, harmonious, and reasonable, while achieving efficiency and fairness. For Jiangxi, Yunnan, Guizhou, and Sichuan, where the income level is relatively backward in economic development, adequate social security should be given to low-income people while stabilizing the proportion of middle-income groups; for Shanghai, Zhejiang, and Jiangsu, provinces with leading and rapid development, publicity can be increased for the third distribution, which is based on the principle of voluntariness and led by individuals or enterprises, and thus in economically developed and relatively wealthy. In this way, regions with advanced economies and relatively concentrated wealth can create a more relaxed institutional environment conducive to the development of philanthropy and the creation of public welfare funds and encourage local affluent people and owners of large enterprises to devote themselves to social welfare.

Strengthen the mechanism of regional industrial synergy and cooperation and lead the region to common prosperity in material life with high-quality economic development. Optimize regional resource allocation, especially strengthening the proper flow of talents and technical resources to the middle and upper reaches of the region. Similarly optimize regional industrial layout, rationalize industrial transfer channels, and improve regional collaborative innovation capacity. It is important to continuously promote high-quality regional industrial development, narrow the gap and unevenness of regional economic development, and then drive regional people to achieve common prosperity in material life.

Deepen the sharing of regional livelihood infrastructure and basic public services to consolidate the basic conditions for social harmony and spiritual enrichment. Increase infrastructure investment in education, pension, and healthcare in Guizhou, Chongqing, Yunnan, and Jiangxi. In addition, empower regional basic public services to co-build and share with digital reform, continuously improve regional public service equalization, and boost regional social harmony and spiritual enrichment. 

Strengthen the ability of collaborative regional environmental management and enhance the livability of the ecological environment. Strengthen ecological environmental protection and increase forest coverage in Shanghai, Jiangsu, Anhui, Sichuan, and other provinces and cities. In addition, increase the area of park green space in Yunnan, Guizhou, Hunan, Jiangxi, and other provinces. Continuously promote the coordination of economic development and ecological environment, rational use of natural resources under the premise of ecological protection, and industrial projects in line with regional characteristics [39]. In the same vein, improve regional ecological and environmental governance education, ecological governance information disclosure, and diversified ecological compensation system, among others, to improve regional environmental collaborative governance capacity and create a high-quality ecological and livable environment [40].

## Figures and Tables

**Table 1:** Evaluation system for the level of common prosperity of the regions along the Yangtze Economic Belt.

Level Indicators	The Secondary Indicators	Symbol
M Material affluence	M_1_ GDP Per Capita (Yuan)	+
	M_2_ The added value of the tertiary industry as a proportion of GDP (%)	+
	M_3_ R&D expenditure as a percentage of GDP (%)	+
	M_4_ Urbanization rate of registered population (%)	+
	M_5_ Consumer Price Index (%)	-
	M_6_ Engel coefficient	-
	M_7_ Disposable income of urban and rural residents	-
S Harmonious social life	S_1_ Basic pension insurance participation rate (%)	+
	S_2_ Number of beds in nursing homes per 1000 permanent residents (beds)	+
	S_3_ Basic medical insurance participation rate (%)	+
	S_4_ Number of beds in medical institutions per 1000 permanent residents (beds)	+
	S_5_ Health technicians per 1000 permanent residents	+
	S_6_ Urban registered unemployment rate (%)	-
	S_7_ Per capita housing area of urban and rural residents (m^2^)	+
	S_8_ The number of household cars per 100 households in cities and towns	+
	S_9_ The number of household cars owned by every 100 rural households	+
C Rich spiritual life	C_1_ Proportion of urban and rural residents’ expenditure on education, culture and entertainment (%)	+
	C_2_ Education spending as a percentage of general public budget spending (%)	+
	C_3_ Number of teachers per 1000 permanent residents	+
	C_4_ The number of students in colleges and universities per thousand permanent residents	+
E Livable ecological environment	E_1_ Green coverage in built-up areas (%)	+
	E_2_ Per capita park green space (m^2^)	+
	E_3_ Forest cover rate (%)	+
	E_4_ Sewage treatment rate (%)	+
	E_5_ Harmless treatment rate of domestic waste (%)	+

**Table 2:** Weight of each indicator in 2019.

Indicator	Weight	Indicator	Weight	Indicator	Weight
$M_{1}$	0.085	$S_{3}$	0.021	$C_{3}$	0.031
$M_{2}$	0.062	$S_{4}$	0.036	$C_{4}$	0.036
$M_{3}$	0.042	$S_{5}$	0.058	$E_{1}$	0.027
$M_{4}$	0.060	$S_{6}$	0.028	$E_{2}$	0.049
$M_{5}$	0.040	$S_{7}$	0.028	$E_{3}$	0.037
$M_{6}$	0.033	$S_{8}$	0.075	$E_{4}$	0.047
$M_{7}$	0.026	$S_{9}$	0.032	$E_{5}$	0.015
$S_{1}$	0.029	$C_{1}$	0.026		
$S_{2}$	0.057	$C_{2}$	0.019		

**Table 3:** Regional common prosperity Index and ranking results along the Yangtze River Economic Belt from 2010 to 2019.

Year	Indicator	Shanghai	Zhejiang	Jiangsu	Anhui	Hubei	Hunan	Jiangxi	Sichuan	Chongqing	Yunnan	Guizhou
2010	Index	0.569	0.697	0.478	0.225	0.362	0.261	0.208	0.307	0.256	0.294	0.119
	Ranking	2	1	3	9	4	7	10	5	8	6	11
2011	Index	0.533	0.598	0.494	0.245	0.345	0.267	0.199	0.317	0.27	0.253	0.381
	Ranking	2	1	3	10	5	8	11	6	7	9	4
2012	Index	0.587	0.652	0.529	0.253	0.35	0.266	0.196	0.324	0.4	0.256	0.129
	Ranking	2	1	3	9	5	7	10	6	4	8	11
2013	Index	0.522	0.671	0.594	0.29	0.337	0.261	0.263	0.347	0.421	0.1	0.135
	Ranking	3	1	2	7	6	9	8	5	4	10	11
2014	Index	0.537	0.658	0.617	0.285	0.371	0.298	0.267	0.421	0.457	0.188	0.223
	Ranking	3	1	2	8	6	7	9	5	4	11	10
2015	Index	0.462	0.671	0.647	0.261	0.332	0.295	0.222	0.377	0.394	0.223	0.141
	Ranking	3	1	2	8	6	7	10	5	4	9	11
2016	Index	0.551	0.646	0.65	0.254	0.336	0.293	0.234	0.405	0.452	0.217	0.158
	Ranking	3	2	1	8	6	7	9	5	4	10	11
2017	Index	0.544	0.629	0.719	0.299	0.364	0.33	0.259	0.412	0.326	0.252	0.184
	Ranking	3	2	1	8	5	6	9	4	7	10	11
2018	Index	0.558	0.601	0.701	0.341	0.402	0.343	0.254	0.403	0.324	0.266	0.203
	Ranking	3	2	1	7	5	6	10	4	8	9	11
2019	Index	0.539	0.593	0.694	0.345	0.403	0.392	0.266	0.433	0.324	0.273	0.213
	Ranking	3	2	1	7	5	6	10	4	8	9	11

**Table 4:** Differences in common prosperity in different river basins from 2010 to 2019.

Region	2010	2011	2012	2013	2014	2015	2016	2017	2018	2019
Upstream Area	0.244	0.305	0.277	0.276	0.322	0.284	0.308	0.294	0.299	0.311
Midstream region	0.264	0.264	0.266	0.288	0.305	0.278	0.279	0.313	0.335	0.352
Downstream area	0.581	0.542	0.589	0.596	0.604	0.593	0.616	0.631	0.620	0.609

**Table 5:** Optimal proximity of the factor of material affluence.

Sample	D+	D-	Statistic Ci	Ranking
Shanghai	0.002	0.117	0.985	1
Zhejiang	0.054	0.065	0.547	3
Jiangsu	0.044	0.077	0.637	2
Anhui	0.098	0.024	0.197	6
Hubei	0.082	0.036	0.306	4
Hunan	0.098	0.024	0.195	7
Jiangxi	0.105	0.017	0.139	9
Sichuan	0.101	0.020	0.169	8
Chongqing	0.082	0.036	0.302	5
Yunnan	0.114	0.007	0.060	10
Guizhou	0.116	0.006	0.050	11

**Table 6:** Optimal proximity of the social life harmony factor.

Sample	D+	D-	Statistic Ci	Ranking
Shanghai	0.088	0.025	0.224	9
Zhejiang	0.035	0.070	0.668	2
Jiangsu	0.018	0.093	0.839	1
Anhui	0.061	0.044	0.417	6
Hubei	0.055	0.050	0.477	5
Hunan	0.060	0.059	0.496	4
Jiangxi	0.079	0.027	0.251	8
Sichuan	0.043	0.073	0.631	3
Chongqing	0.094	0.013	0.125	11
Yunnan	0.082	0.040	0.330	7
Guizhou	0.090	0.022	0.193	10

**Table 7:** Optimal proximity of the spiritual life abundance factor.

Sample	D+	D-	Statistic Ci	Ranking
Shanghai	0.165	0.016	0.081	11
Zhejiang	0.095	0.073	0.432	6
Jiangsu	0.023	0.164	0.872	1
Anhui	0.068	0.123	0.640	4
Hubei	0.081	0.089	0.527	5
Hunan	0.058	0.115	0.665	3
Jiangxi	0.106	0.062	0.369	8
Sichuan	0.0561	0.118	0.678	2
Chongqing	0.135	0.035	0.204	10
Yunnan	0.107	0.070	0.397	7
Guizhou	0.123	0.051	0.295	9

**Table 8:** Optimal proximity of ecological environment livable factor.

Sample	D+	D-	Statistic Ci	Ranking
Shanghai	0.066	0.100	0.602	3
Zhejiang	0.042	0.084	0.664	2
Jiangsu	0.074	0.062	0.457	5
Anhui	0.089	0.031	0.258	11
Hubei	0.072	0.049	0.406	7
Hunan	0.094	0.062	0.396	8
Jiangxi	0.077	0.070	0.473	4
Sichuan	0.076	0.046	0.376	10
Chongqing	0.021	0.102	0.831	1
Yunnan	0.010	0.064	0.392	9
Guizhou	0.081	0.064	0.441	6

**Table 9:** Classification of common prosperity and development level in various regions.

Level of Common Prosperity	Included Areas
High-level common prosperity	Zhejiang, Jiangsu, Hubei
Unbalanced development	Shanghai, Anhui, Sichuan, Hunan, Chongqing
Low-level common prosperity	Jiangxi, Yunnan, Guizhou

## Data Availability

No applicable.

## References

[1] Xi J.P. (2021). Speech at the National Poverty Alleviation Summary and Commendation Conference. People’s Daily.

[2] Duan J. (2017). Exploring the path to solve the problem of the gap between the rich and the poor in the process of China common prosperity. Teach. Res..

[3] The Xinhua News Agency. http://www.gov.cn/xinwen/2020-11/15/content_5561711.htm.

[4] Wang Y.Z., Duan X.J., Wang L. (2020). Spatial-temporal differentiation and driving mechanism of economic development in the Yangtze river economic belt. Resour. Environ. Yangtze Basin.

[5] Li T.Z., Yang W.J., Li W.Y. (2021). Research on optimal allocation of higher education resources in the Yangtze River Economic Belt. China High. Educ. Res..

[6] Wang S.W., Xu L.J., Zhou W.H. (2021). Evaluation of scientific competitiveness and regional heterogeneity of the Yangtze River Economic Belt. Sci. Technol. Prog. Policy.

[7] Peng X.J., Cheng J.H., Fang C.D. (2021). Study on coordinated development of economy-resources-environment in Yangtze River Economic Belt based on “Three lines and one single order”. China Popul. Resour. Environ..

[8] Karl M., Frederick E. (1980). Karl Marx Frederick Engels Collected Works.

[9] Li S., Chen Z.S., Shi J.C., Liu T.F., He W.J. (2022). A written talk on the theme of “Common Prosperity”. J. Zhejiang Univ..

[10] Karl M., Frederick E. (1995). Karl Marx Frederick Engels Collected Works.

[11] Karl M., Frederick E. (2009). Karl Marx Frederick Engels Collected Works.

[12] Jiang Y.M., Xie Q. (2021). Solid promotion of common prosperity: Logical path and realization path. Econ. Rev..

[13] Deng X.P. (1993). Anthologies of Deng Xiaoping.

[14] Jiang Z.M. (2006). On Socialist Market Economy.

[15] Cao Y.X., Liu Y.M. (2019). Common prosperity and its realization path in the new era. Theor. J..

[16] The people’s Government of the People’s Republic of China Portal. http://www.gov.cn/gongbao/content/2021-5/20/content_5621189.htm.

[17] Song Q.A. (2014). Preliminary study on the connotation, characteristics and evaluation indexes of common prosperity in China. Globalization.

[18] Liu P.L., Qian T., Huang X.H. (2021). The connotation, realization path and measurement of common prosperity. Manag. World.

[19] Yu J.X., Ren J. (2021). The theoretical connotation and policy agenda of common prosperity. Cass J. Political Sci..

[20] Zhang L.M., Li J.W. (2021). The connotation, strategic Objectives and policy measures of promoting common prosperity. Reform.

[21] Germano F. (2022). Entropy, directionality theory and the evolution of income inequality. J. Econ. Behav. Organ..

[22] Reindl I., Tyran J.R. (2021). Equal opportunities for all? How income redistribution promotes support for economic inclusion. J. Econ. Behav. Organ..

[23] Welsch H., Bonn U. (2008). Economic convergence and life satisfaction in the European Union. J. Socio-Econ..

[24] Wan H.Y., Chen J.P. (2021). The theoretical connotation and quantitative method of common prosperity. Financ. Trade Econ..

[25] Jiang Y.M., Dou X.L. (2022). Solid promotion of common prosperity index System construction: Theoretical logic and Preliminary Design. Southeast Acad. Res..

[26] Yang Y.Y., Wang M.J. (2021). Common prosperity: Evolution course, Stage goal and evaluation system. Jianghai J. Res..

[27] Duarte R., Riazuelo Á.G., Sáez L.A., Sarasa C. (2022). Economic and territorial integration of renewables in rural areas: Lessons from a long-term perspective. Energy Econ..

[28] Jia Z., Lin B. (2021). How to achieve the first step of the carbon-neutrality 2060 target in China: The coal substitution perspective. Energy.

[29] Chen Y.S., Zhang S.H., Hu D.S., Lian L.B. (2017). The development of China’s Yangtze River Economic Belt: How to make it in a green way?. Sci. Bull..

[30] Yan H.J., Hu X.F., Wu D.W., Zhang J.N. (2021). Exploring the green development path of the Yangtze River Economic Belt using the entropy weight method and fuzzy-set qualitative comparative analysis. PLoS ONE.

[31] Chen L.J., Yu J.X., Xu Y. (2021). The construction of common prosperity index model. Gov. Res..

[32] Shen Y., Li J.R. (2020). Research on the evaluation index system of Rural residents’ living affluence in China: Based on the perspective of building a moderately prosperous society in an all-round way. World Surv. Res..

[33] Fang X.P., Liao X.L., Deng Y.J. (2020). Measurement of high quality development level of China’s provinces. Stat. Decis..

[34] Zhang R.J., Dong H.Z. (2022). Urban green development level measurement and spatial correlation structure analysis in the Yangtze River Economic Belt. Stat. Decis..

[35] Xi J.P. (2021). Solid promotion of common prosperity. Pract. Ideol. Theor. Ed..

[36] Chen J.M., Huo Z.H. (2020). Evaluation and comparison of regional green development level along the Yangtze river economic belt. Sci. Technol. Manag. Res..

[37] Kacprzak D. (2017). Objective Weights Based on Ordered Fuzzy Numbers for Fuzzy Multiple Criteria Decision Making Methods. Entropy.

[38] Kacprzak D. (2019). A doubly extended TOPSIS method for group decision making based on ordered fuzzy numbers. Expert Syst. Appl..

[39] Li M.J., Chen G.H., Lin Z.B. (2015). Research on dynamic evaluation method based on ideal solution. Chin. J. Manag. Sci..

[40] Qiu H.P. (2016). The scientific connotation and realization way of common prosperity. China Rev. Political Econ..

